# List of Diseases at the Bath City Dispensary, from June 1, to July 1, 1802

**Published:** 1802-09-01

**Authors:** 


*List of Diseases at the Bath City Dispensary, from June* 1, *to July* 1, 1802.

| | | | |
|---|---|---|---|
| Asthma | 3 | Menorrhagia | 1 |
| Abscess | 1 | Phthisical | 6 |
| Anasarca | 5 | Pulmonary Complaints | 3 |
| Contusion | 1 | Peripneumonia Notha | 1 |
| Cholera | 4 | Paralysis | 1 |
| Colica | 2 | Pleurodyne | 1 |
| Chlorosis | 2 | Physconia | 1 |
| Diarrhœa | 2 | Psora | 3 |
| Dyspepsia | 1 | Rheumatismus | 8 |
| Eruptiones | 4 | Rubeola | 2 |
| Erythema | 1 | Synochus Biliosa | 14 |
| Fever (Simple) | 7 | Syphilis | 3 |
| Febrile Complaints | 4 | Shingles | 1 |
| Gastrodynia | 2 | Spleen Enlarged | 1 |
| Gravelly Complaints | 5 | Strain | 1 |
| Hernia | 3 | Tussis | 3 |
| ——— Humoralis | 2 | Typhus | 3 |
| Hemicrania | 1 | Urticaria | 1 |
| Hæmorrhagia | 1 | Ulcers | 8 |
| Hæmatemesis | 1 | Vermes | 1 |
| Herpes | 1 | | |
| Hydrocele | 1 | Total | 119 |
| Infantile Complaints | 2 | | |

*Ditto from July* 1 *to August* 1, 1802.

| | | | |
|---|---|---|---|
| Angina | 1 | Cynanche Maligna | 1 |
| Asthma | 2 | ——— Parotidæa | 1 |
| Abscess | 3 | Cholera | 2 |
| Anasarca | 3 | Colica | 3 |
| Bowel Complaints | 5 | ——— Pictonum | 1 |

Chlorosis

| | |
|---|---|
| Chloroſis | 4 |
| Contuſion | 1 |
| Dyſenteria | 1 |
| Diarrhœa | 3 |
| Dyſpepſia | 2 |
| Eryſipelas | 1 |
| Eruptiones | 4 |
| Febris Simplex | 5 |
| —— interm. quotid. | 2 |
| Gaſtrodynia | 6 |
| Hernia Humoralis | 1 |
| Herpes | 2 |
| Hyſteria | 1 |
| Inflammation | 1 |
| Icthyoſis | 1 |
| Lepra | 2 |
| Nephritic Complaints | 4 |
| Neuroſis | 2 |
| Ophthalmia | 1 |
| Phthiſis Pulmonalis | 4 |
| Pulmonary Complaints | 2 |
| Paraphymoſis | 1 |
| Pleurodyne | 4 |
| Pſora | 4 |
| Rheumatiſmus | 12 |
| Rubeola | 7 |
| Synochus Bilioſa | 13 |
| —— Pleuritica | 1 |
| —— Cholerica | 1 |
| —— Dyſenterica | 1 |
| Syphilis | 3 |
| Scrofula | 3 |
| Tuſſis | 1 |
| Tinea | 1 |
| Ulcerated Legs | 4 |
| Variola Conferta | 1 |
| —— Diſcreta | 3 |
| Total | 132 |

---

*Bath City Diſpenſary, June* 28, 1802.

THE Committee, upon the repreſentation of the Phyſicians of this Inſtitution, being perſuaded that the general extenſion of Vaccine Inoculation amongſt the children of the poor, would effectually diminiſh the great mortality occaſioned by Small-pox in this city, give notice, that all perſons applying at the Diſpenſary may be inoculated without recommendation or expence. Attendance for this purpoſe will be given at the Diſpenſary every Saturday, by Mr. Creaſer and Mr. White, at twelve o'clock; and the Committee cannot too ſtrongly exhort the poorer claſſes of this city to avail themſelves of this advantage, as the adoption of Vaccine Inoculation, when placed in their power, is a duty which they owe to themſelves and others.

In conſequence of the circulation of the above notice, 33 children have been inoculated ſince July 3.

*Observations*

